# Impact of Harvest Maturity and Controlled Atmosphere on Strawberry Quality Under Simulated Export Conditions

**DOI:** 10.3390/foods14172959

**Published:** 2025-08-25

**Authors:** Hyang Lan Eum, Ji-Hyun Lee, Jeong Gu Lee, Min-Sun Chang, Kyung-Ran Do, Haejo Yang, Kang-Mo Ku, Dong-Shin Kim

**Affiliations:** 1 Highland Agriculture Research Institute, National Institute of Crop and Food Science, Pyeongchang-Gun 25342, Republic of Korea; 2 Postharvest Research Division, National Institute of Horticultural and Herbal Science, Wanju 55365, Republic of Korea; leejh80@korea.kr (J.-H.L.); ljg89@korea.kr (J.G.L.); aeru@korea.kr (M.-S.C.); microdo@korea.kr (K.-R.D.); gowh1231@korea.kr (H.Y.); 3 Department of Plant Biotechnology, College of Life Sciences and Biotechnology, Korea University, Seoul 02841, Republic of Korea; ku_km@korea.ac.kr

**Keywords:** firmness, respiration, decay, metabolite profiling, VOCs, lipid peroxidation

## Abstract

This study aimed to evaluate the effects of controlled atmosphere (CA) treatment on the postharvest quality of strawberries harvested at different 50% and 80% maturity under export shipping conditions. The strawberries were subjected to CA and refrigerated container (Reefer) environments at 10 °C, and their quality attributes were then analyzed. Metabolomic profiling revealed significant variations in primary and secondary metabolites and volatile organic compounds (VOCs). A pathway analysis revealed that CA conditions altered metabolic pathways related to sugar, amino acid, and energy metabolism during storage. CA treatment effectively delayed the accumulation of anthocyanins and enhanced the levels of specific amino acids and VOCs essential for the flavor and aroma of strawberries. Bioluminescence imaging revealed that CA treatment effectively reduced lipid peroxidation. A correlation analysis showed that certain VOCs and secondary metabolites significantly correlated with lipid peroxidation, indicating their role in enhancing antioxidant activity and reducing oxidative stress. These results suggest that CA conditions are associated with significantly reduced weight loss, the maintenance of firmness, and lower respiration rates in strawberries, particularly in those harvested at 80% maturity, extending the shelf life and improving the sensory quality of strawberries. Therefore, CA treatment is an effective method for long-term export.

## 1. Introduction

Strawberry (*Fragaria* × *ananassa* Duch.) is a major global horticultural commodity, attracting interest for its sweet flavor and taste. The global fresh strawberry market was USD 19 billion in 2024 and is projected to grow to USD 31.82 billion by 2034 [1,2]. In South Korea, strawberries represent a major export crop, with production reaching 159,000 tons and exports exceeding USD 69 million in 2023 [3]. While approximately 96% of strawberries exported from Korea were transported via air, support for the costs of export logistics was discontinued in 2024. Owing to the higher logistics costs associated with air cargo pallet transport compared with those of sea freight transport for heavier agricultural products, ships must be used for exports. The containers primarily used for shipping agricultural products are refrigerated intermodal transport containers, commonly known as reefer containers, which are essential in the global food supply chain [4,5]. Most reefer containers only regulate temperature; however, approximately 15% are controlled atmosphere (CA) containers in which the internal environment (O_2_ and CO_2_) is adjusted to minimize respiration and enable long-term transport [5].

Strawberries (*Fragaria × ananassa*) are highly perishable non-climacteric fruits, and their limited postharvest life poses significant challenges for export. The shelf life of fresh strawberries is typically around 7 days and is further shortened when the fruit is physically damaged [6]. To extend marketability during long-distance distribution, particularly to countries such as Vietnam and Hong Kong, Korean strawberries are often harvested at an early stage with less than 50% surface coloration (Figure S1). These fruits gradually attain approximately 80% coloration during transit. However, due to the non-climacteric nature of strawberries, early harvesting prior to physiological maturity results in the insufficient development of key sensory attributes, including sweetness and aroma [7,8]. Consequently, strawberries that ripen off the plant tend to be inferior in flavor compared to those ripened on the mother plant, leading to decreased consumer satisfaction in importing markets [9]. In this study, fruit skin coloration was used as the primary index for harvest maturity. To preserve the intrinsic flavor and enhance the export quality, harvesting at approximately 80% coloration on the mother plant is proposed. While this stage is associated with an increased risk of softening, the application of appropriate postharvest technologies to mitigate texture degradation could enable an extended shelf life and maintain marketability in destination countries. Strawberry cultivation in Korea typically begins with harvesting in December and continues until May. In Korea, strawberries cultivated during the winter season are typically June-bearing cultivars grown under protected greenhouse conditions with an artificially controlled temperature and photoperiod. This production system begins with the first harvest in December and continues until May of the following year, making it well-suited to the low-temperature conditions of winter. However, as ambient temperatures rise in May, the rapid softening and excessive coloration of the fruit often occur, resulting in a decline in marketability [10].

Precooling, high-CO_2_ treatment, and modified atmosphere packaging (MAP) storage, among other methods, have been used to extend the shelf life of strawberries by maintaining their physiological and biochemical characteristics [7,10,11]. Temperature is the most significant factor affecting the quality of perishable fruits. Precooling minimizes respiration and moisture loss, thereby helping to maintain quality during distribution [6]. Modified atmosphere conditions, such as MAP or CA storage, help to control the gas composition to slow respiration and microbial growth; an MAP with 11–14% O_2_ and 9–12% CO_2_ extends the aroma and shelf life of strawberries [12,13], while short-term high-CO_2_ treatment reduces decay and maintains firmness [7,11,14]. The treatment of strawberries with 30% CO_2_ for approximately 3 h can effectively maintain the firmness of strawberries and reduce mold growth [11].

The quality of strawberries is significantly influenced not only by primary metabolites such as sugars and organic acids [15] but also by secondary metabolites including anthocyanins, flavonoids, and phenolic acids. These compounds contribute to the taste, aroma, nutritional value, and resistance to postharvest stress, making them critical for maintaining fruit quality and meeting consumer preferences during distribution and storage. Among postharvest environmental factors, the O_2_ and CO_2_ are known to profoundly affect the metabolic pathways associated with the synthesis and degradation of these metabolites. CA storage, in particular, has been reported to modulate sugar metabolism [16,17], delay fruit softening, and influence the accumulation of anthocyanins and other phenolic compounds [18,19,20].

The aroma of strawberries, determined by a complex mixture of more than 360 compounds—including esters, aldehydes, ketones, alcohols, acids, and terpenes—is highly sensitive to postharvest environmental conditions [21,22]. Among these volatiles, 15–25 volatile compounds, particularly methyl and ethyl esters, furanones, C6 aldehydes, and C6 derivatives, contribute significantly to the strawberry aroma. During storage, however, off-flavor-related metabolites, such as acetaldehyde, ethanol, and ethyl acetate, are produced, negatively affecting the taste and overall quality of strawberries [23].

Bioluminescence measurement using an In Vivo Imaging System (IVIS) provides a nondestructive technique for assessing the fruit quality [24,25]. ROS generated during fruit aging induce lipid peroxidation, resulting in low-intensity photon emissions detectable via IVIS [26]. These reactions result in bioluminescence, which can be detected using a charge-coupled device (CCD)-based IVIS system [24,25].

Bioluminescence measurement was employed for the nondestructive determination of strawberry quality [24] and the identification of the tomato-ripening stages [25]. The application of IVIS in CA-treated strawberries may provide insight into oxidative stress and membrane stability under different storage conditions.

Therefore, this study aimed to evaluate the efficacy of CA containers in preventing the quality degradation of strawberries, picked at different maturity stages (50% and 80%), during their long-term transportation by ship. To this end, we assessed the quality attributes, physicochemical properties, and metabolite composition of strawberries, and analyzed the bioluminescence using IVIS to investigate the effects of CA on strawberry quality during maritime export.

## 2. Materials and Methods

### 2.1. Plant Material and Container Conditions

Strawberries (*Fragaria × ananassa* Duch. cv. Geumsil), the most widely exported Korean cultivar to Southeast Asia, were harvested from a farm in Jinju, Gyeongsangnam-do, Korea, on 17 March 2023. The fruit was picked early in the morning and promptly transported to a local cooperative sorting facility. This cultivar was selected for the study due to its strong representation of Korea’s export market and frequent issues related to insufficient ripening at the time of export. Strawberries with an initial internal temperature of approximately 23 °C were subjected to room cooling in a cold chamber set at 1 °C. The fruits were cooled until reaching 7/8 of the target transport temperature (4 °C), which required approximately 15 h. After precooling, strawberries without visible defects (e.g., cracks, rot, malformations, or mechanical damage) were selected and classified into two maturity groups based on external skin coloration: 50% and 80%. The maturity stages of strawberry fruits were determined based on the extent of external skin coloration. Strawberries at 50% maturity were defined as those with red pigmentation extending from the distal end up to approximately halfway toward the calyx (i.e., 50% of the surface from tip to calyx showing red coloration), whereas 80% maturity was defined as at least 80% of the fruit surface exhibiting uniform red color. The classification was visually conducted by two trained evaluators after precooling. Representative images for each stage are provided in Figure S1.

Strawberries were packed in transparent plastic clamshell boxes made of polyethylene terephthalate (PET, thickness: 0.30 mm; 330 g per box), with four clamshells placed into each corrugated cardboard box. The packaged boxes were transported to the laboratory in refrigerated vans maintained at 4 °C. Strawberries were subjected to a simulated maritime export procedure designed to replicate real export logistics conditions. The fruits were transported for 7 days at 4 °C using either a conventional reefer container (DAIKIN Industries Ltd., Osaka, Japan) serving as the control or a controlled atmosphere (CA) container (Star Cool Integrated, Maersk Container Industry, Tinglev, Denmark) as the treatment. Both systems utilized 20-foot containers (6.1 × 2.4 × 2.6 m^3^). For the CA container, a CA curtain (CA curtain kit incl. 5 tools 818286A, Star Cool Integrated) was installed immediately after loading to minimize gas leakage through the door gaskets. The CA container operated as an active atmosphere control system. Initially, oxygen concentration was reduced using a nitrogen generator (EsporaLab Co., Yangju,, Republic of Korea) supplying nitrogen-rich air at a flow rate of 530 L/min. Carbon dioxide was then injected to achieve and maintain the target atmosphere of 5% O_2_ and 12% CO_2_. These conditions were continuously monitored throughout the 7 days transport period using a built-in real-time environmental control system capable of logging internal temperature, relative humidity (RH), and CO_2_ concentration. Moreover, the CA container was completely sealed to block any external air exchange, which also enabled the maintenance of a relatively high RH (≥90%). During the entire transport period, the interior of both container types was maintained in complete darkness. To assess the microclimate within the retail packaging, the temperature and RH inside individual clamshells were monitored using a WatchDog Model B102 T/RH data logger (Spectrum Technologies Inc., Aurora, IL, USA).

Following the 7 days simulated maritime transport, the containers were opened, and the strawberries were immediately transferred to a cold storage facility maintained at 10 °C. This step simulated domestic distribution environments after arrival. Cold chain continuity was strictly maintained during the entire transition and storage process. The fruits were held for an additional 1, 3, or 7 days at 10 °C, referred to as 7 D/1 d, 7 D/3 d, and 7 D/7 d, respectively, to simulate varying distribution periods under realistic conditions.

### 2.2. Quality Evaluation

After the 7 D simulated transport in the container and cold distribution (7 D/1 d, 7 D/3 d, and 7 D/7 d), the weight loss percentage, firmness, color index (CIE L*, a*, b*, chroma, and hue angle), decay rate, and respiration rate during cold distribution were observed. The weight change was expressed as a percentage of the initial weight of each clamshell box during cold distribution (*n* = 3). Firmness was measured in Newton (N) using a texture analyzer (TA.XT2, Stable Micro Systems Texture Technologist) with a 5 mm probe at a speed of 120 mm/s in the equatorial region of the fruit (*n* = 40). Color was measured at two points in the equatorial region using a colorimeter (CR 400; Minolta, Osaka, Japan) (*n* = 10). The respiration rate was determined by placing 100–110 g of strawberries in a 500 mL sealed container, sealing it for 1 h, and extracting 1 mL of gas from the container headspace using a gastight syringe. The gas was analyzed using a gas chromatograph (Agilent 6890A GC System; Agilent Technologies, Santa Clara, CA, USA). The CO_2_ accumulated in the container was detected using a thermal conductivity detector (column: 100 °C; detector: 120 °C; carrier gas: He at 30 mL/min) with a packed stainless-steel column. The decay rate was expressed as the percentage of decayed fruits relative to the total number of fruits in each clamshell box (*n* = 6).

### 2.3. Sample Preparation for Quantitation of Amino Acids and Metabolites

The metabolite analysis was conducted with five replicates, each consisting of 10 strawberries. Each biological replicate was composed of 10 pooled strawberries, which were mixed, frozen in liquid nitrogen, and stored at −80 °C for further analysis. The frozen samples were freeze-dried before analyzing amino acids and untargeted metabolites.

### 2.4. Analysis of Free Amino Acids

For extracting free amino acids, 1 g of freeze-dried strawberry sample was mixed with 50 mL of an extraction buffer composed of 0.1 M perchloric acid and 0.1% metaphosphoric acid in triple-distilled water. The mixture was sonicated for 1 h to facilitate extraction. After sonication, the mixture was left at room temperature and agitated at 150 rpm for an additional 1 h to ensure thorough mixing. The extract was then filtered using a 0.2 μm syringe filter to remove any particulate matter. The filtered extract was analyzed using high-performance liquid chromatography (HPLC). The separation was performed on an Inno C18 column (150 mm × 4.6 mm, 5 μm, YoungJin Biochrom, Seongnam, Republic of Korea). The mobile phase consisted of two components: mobile phase A, which was 40 mM sodium phosphate dibasic, pH 7.0, and mobile phase B, which was a mixture of triple-distilled water, acetonitrile, and methanol in a 10:45:45 volume ratio. The injection volume was 0.5 μL and the elution was performed at a flow rate of 1.5 mL/min. The elution gradient was programmed as follows: 5% mobile phase B at 0 min, maintained for 3 min, gradually increased to 55% B by 24 min, and further increased to 80% B by 25 min. This condition was held until 31 min, followed by a return to 5% B at 34.5 min, and maintaining it until the end of the run at 35 min. The column temperature was set at 40 °C, and the amino acids were detected at 338 nm.

### 2.5. Gas Chromatography Mass Spectrometry (GC-MS) of Polar Phase Metabolites

Polar phase metabolites were analyzed using a modified version of the method described by Hyun et al. [27]. A 0.05 g sample of freeze-dried material was extracted with 1 mL of 80% methanol via sonication at 60–70 °C for 30 min. The extract was centrifuged at 15,000× *g* for 10 min at 4 °C, and 700 μL of the supernatant was collected. To this supernatant, 500 μL of chloroform, 20 μL of ribitol (internal standard, IS), and 700 μL of distilled water (DW) were added, and the mixture was vortexed. The mixture was then centrifuged at 2500× *g* for 10 min. Subsequently, 500 μL of the supernatant was concentrated at room temperature under reduced pressure using a nitrogen evaporator (EvaT-0200; Goojung Engineering Co., Seoul, Republic of Korea).

The concentrated sample was redissolved in 50 μL of methoxyamine hydrochloride (10 mg/mL in pyridine) and incubated in the dark at 37 °C for 2 h. Following the reaction, 40 μL of the sample was transferred to a vial, and 100 μL of N-methyl-N-trifluoroacetamide was added to it. The mixture was incubated in the dark at 37 °C for 30 min and analyzed using a GC-MS ISQ LT system (Thermo Fisher Scientific, Waltham, MA, USA). For the GC-MS analysis, the oven temperature was programmed to increase from 50 °C to 310 °C at a rate of 5 °C/min. The injector was set to a splitless mode at 250 °C. Helium was used as a carrier gas at a flow rate of 1 mL/min. The column used was a DB-5-fused silica capillary column (0.25 mm × 30 m × 0.25 μm, Agilent, Santa Clara, CA, USA). The mass scan (*m*/*z*) range was set from 35 to 550.

### 2.6. Ultra-Performance Liquid Chromatography (UPLC)–Quadrupole Time-of-Flight (QTOF)–MS Analysis of Metabolites

For extracting strawberry metabolites, freeze-dried samples (0.01 g) were homogenized in 70% aqueous methanol (*v*/*v*) containing an IS (zidovudine) using a bullet blender. After centrifugation (12,000× *g*, 10 min, 5 °C), 1 μL of clear supernatant was injected into an Acquity BEH C18 column (2.1 mm × 100 mm, 1.7 μm; Waters) coupled with UPLC-QTOF MS (XevoTM G2-S; Waters, Milford, MA, USA). The column was equilibrated with solvent A [water containing 0.1% formic acid (FA)] at a flow rate of 0.35 mL/min at 40 °C, and the metabolites were eluted with a linear gradient of solvent B (acetonitrile containing 0.1% FA) for 8 min. The eluted metabolites were ionized using electrospray ionization in the negative mode and detected using a QTOF MS system. Leucine–enkephalin ([M + H] = 554.2615 Da) was used as a lock mass reference compound for exact mass measurements. MS data were obtained over an *m*/*z* range of 50–1200, and MS/MS spectra were obtained using collision energy ramps of 10–30 or 20–40 eV [27]. The data obtained using UPLC-QTOF MS were aligned using the MassLynx software 4.2. (Waters) and normalized to the IS. Tentative identification of metabolites was performed using the ChemSpider database in UNIFI software (Waters) and the METLIN database (www.metlin.scripps.edu (accessed on 4 September 2024).

### 2.7. Analysis of Volatile Organic Compounds

Volatile organic compounds (VOCs) were analyzed using the headspace method following the procedure described by Jee et al. [28]. The VOCs were collected in thermal desorption (TD) tubes (Tenax TA Cond/Capped Pk 10, C1-AAXX-5003, Markes International, CA, USA). A 100 ± 5 g sample of strawberries was placed in a nonreactive polyethylene bag (200 mm × 200 mm) and thoroughly crushed manually. The bag containing the crushed strawberries was placed inside a magenta box (polycarbonate, W75 mm × D75 mm × H100 mm). The opening of the magenta box was sealed with PVC wrap, and a lid equipped with gas-sampling plastic tubing was securely fitted. The samples were incubated at 30 °C for 20 min. The VOCs collected in the magenta box were sampled by connecting a TD tube to plastic tubing (Tygon S3 E-3603, Tygon S3™, Solon, OH, USA) attached to a pump (Mini pump MP-∑30KNII, Taewon SIBATA Co., Seoul, Republic of Korea). The VOCs were collected at a flow rate of 0.15 L/min for 2 min.

3-Octanone (Sigma-Aldrich Co., St. Louis, MO, USA) was used as an IS and dissolved in methanol at 25 μg/mL. After collecting the VOCs, 4 μL of the IS was injected into the tube, and a Solution Loading Rig (Markes International, Gold River, CA, USA) was used to evaporate the solvent and prepare the sample for analysis. The sample tube was loaded into a TD-100-xr thermal desorption unit (Thermal Desorption Unit, Markes International, Gold River, CA, USA) for analysis. The sample desorption conditions are as described below. The pre-desorption phase included a pre-purge time of 1 min with a split flow rate of 20 mL/min. The primary tube desorption was conducted at 240 °C for 5 min at a trap flow of 40 mL/min. The sample tube was fully desorbed onto a cold trap (U-T11GPC-2S, Markes International, Gold River, CA, USA) maintained at 25 °C, using a splitless desorption method. The cold trap was heated at 25 °C/s to 270 °C and held at this temperature for 5 min. During trap desorption, the samples were split at a ratio of 6:1. The analysis was performed using a GC-MS-QP 2020 NX system (Shimadzu, Kyoto, Japan). The GC oven, equipped with a DB-5MS capillary column (30 m × 0.25 mm i.d., 0.25 μm film thickness, Agilent Technologies, Santa Clara, CA, USA), was initially maintained at 35 °C for 3 min, then ramped at 6 °C/min to 170 °C, and the temperature was further increased at 12 °C/min to 280 °C. The total runtime was 34 min. The MS ion source and interface temperatures were maintained at 250 °C, and ions were acquired over an *m*/*z* range of 40–400. Peaks in the GC-MS chromatogram were identified by comparison with the NIST17 library.

Peak interpretation was performed using the Automated Mass Spectral Deconvolution and Identification System (AMDIS) software (version 2.73, NIST, Gaithersburg, MD, USA) with specific parameters applied.

### 2.8. Evaluation of Lipid Peroxidation Using IVIS

Strawberries were analyzed for lipid peroxidation using an IVIS III system (PerkinElmer, Waltham, MA, USA) at three time points—harvest, after 7 d (7 D) of simulated container transport, and throughout cold distribution (7 D/3 d and 7 D/7 d). Before evaluation, fruits were preadapted to complete darkness in a dark chamber for 1.3 h. Strawberry specimens were detected and visualized at each time point. Luminescent image data were processed using a program sequence setup for autoluminescence over 20 min with an emission range of 640–770 nm and an excitation block. The exposure time was set to 300 s, with medium binning and an F-stop of 8. The optical luminescent image data were displayed in pseudocolors to represent the intensity. The measurements were repeated three times with different fruits, and the signal intensity of each optical image was calculated within the regions of interest ). The radiance was summed and presented as the average total flux (W/m^2^/s/steradian) with standard deviation.

### 2.9. Statistical Analysis

The experiment was conducted using a completely randomized design. The data are expressed as the mean ± standard deviation. Statistical analyses were performed using ANOVA in SAS (version 9.1). The significance of each measurement was determined using Duncan’s multiple range test at a significance level of *p* < 0.05. Multivariate statistical analysis of the datasets between treatment groups was performed using SIMCA-P+ v.16 (Umetrics, Umeå, Sweden), and visualization was achieved via partial least squares discriminant analysis (PLS-DA). Pathway analysis was conducted using MetaboAnalyst 5.0 https://www.metaboanalyst.ca (accessed on 20 November 2024), based on normalized intensities of identified metabolites and the KEGG database. Pearson’s correlation coefficients were calculated using GraphPad Prism 9.0 (GraphPad Software, San Diego, CA, USA).

## 3. Results and Discussion

### 3.1. Quality Characteristics According to the Maturity Stage and Container Environment

**Figure 1:**
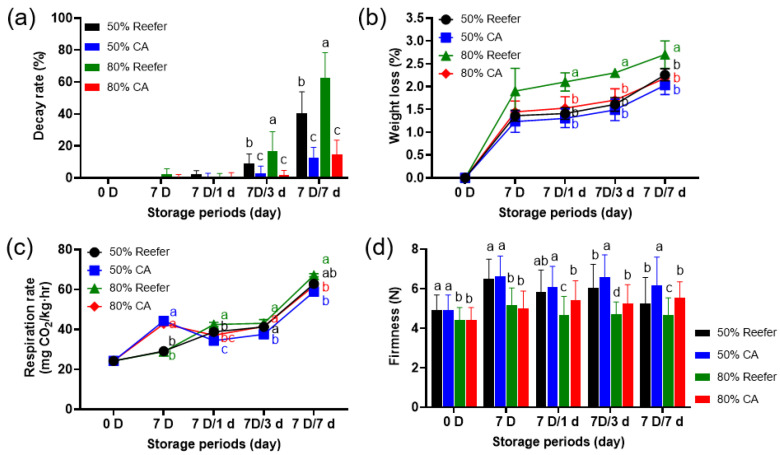
Comparison of the effect of controlled atmosphere (CA; 5% O_2_ + 12% CO_2_) and Reefer (air) container treatments on weight loss (**a**), firmness (**b**), respiration rate (**c**), and decay rate (**d**) of strawberries. The strawberries were harvested (0 D) at different maturity stages (50 and 80%), containerized for 7 days (7 D) at 4 °C, and subjected to cold distribution for 7 days (7 D/1 d, 7 D/3 d, and 7 D/7 d) at 10 °C. Data are expressed as means ± standard deviations. Different letters indicate statistically significant differences.

Strawberries harvested at different maturity stages were treated in a CA or Reefer environment using an export shipping container and then stored at 10 °C. The weight loss increased during the 7 D CA treatment and the subsequent 7 days cold distribution (7 D/7 d) (Figure 1a). The 80%-maturity Reefer group showed the highest weight loss (> 2.8%), whereas no significant differences were observed among the 80%-maturity CA, 50%-maturity Reefer, and 50%-maturity CA treatment groups (*p* < 0.05). Interestingly, CA treatment was more effective in reducing weight loss in more mature strawberries. Several studies have reported a delay in weight loss due to the high CO_2_ concentrations [11,16]. Low O_2_ and high CO_2_ conditions help control the respiration rate of fruits, reducing the moisture loss. This reduction in transpiration and evaporation via respiration helps maintain the internal moisture content, reducing the weight loss rate.

Strawberries soften easily during distribution, limiting their export due to the rapid quality deterioration. The CA treatment also affected the firmness of strawberries (Figure 1b). The firmness of strawberries at 50% maturity was maintained at a higher level than that at 80% maturity, and both the Reefer and CA container treatments had similar effects on the firmness. However, as the cold distribution period progressed, the firmness of strawberries in the Reefer treatment was significantly lower than that in the CA treatment (*p* < 0.05), which was consistent in the 50%- and 80%-maturity groups.

Treating strawberries with a 30% higher concentration of CO_2_ for 3 h effectively maintains firmness [11]. Ripening weakens cellular adhesion due to cell wall structure changes and middle lamella dissolution, leading to a loss of firmness [29,30]. The primary cell-wall-degrading enzymes that reduce cell adhesion are polygalacturonase (PG), pectin methylesterase (PME), pectate lyase (PL), beta-galactosidase, cellulase, and hemicellulose [29,30,31]. Pectin is one of the primary components of the cell wall; its degradation leads to structural changes in the cell wall, altering the texture of the fruit, and ultimately promoting softening [32]. PG degrades galacturonic acid in pectin, weakening the cell adhesion and accelerating softening [30,31,32]. PME demethylates pectin, increasing its water affinity and vulnerability to the action of pectin-degrading enzymes, such as PG and PL [11]. CO_2_ regulates the activity of these enzymes. Being soluble, CO_2_ dissolves easily within the cell and combines with water to form carbonic acid (H_2_CO_3_). Carbonic acid dissociates into hydrogen ions (H^+^) and bicarbonate ions (HCO_3_^−^), altering the intracellular pH [33]. An increase in the H^+^ concentration acidifies the intracellular pH, reducing the activity of cell-wall-degrading enzymes under acidic conditions, which can delay softening [33,34]. Conversely, HCO_3_^−^ can be absorbed by the cell, neutralizing the H^+^ concentration and increasing the intracellular pH. A higher apoplastic pH enhances pectin cross-linking with Ca^2+^, reinforcing the cell wall [34]. In this study, the firmness of strawberries was effectively maintained in CA containers. Thus, 12% CO_2_ and 5% O_2_ preserved firmness by inhibiting cell-wall-degrading enzymes and stabilizing the pectin structure.

The CA condition reduced respiration rates in the 50%- and 80%-maturity strawberries during the subsequent 7 days cold distribution period (7 D/7 d) (Figure 1c). Immediately following CA treatment, respiration rates (43–44 mg CO_2_/kg·h) were higher than in Reefer samples (28–29 mg CO_2_/kg·h), regardless of maturity. However, during cold distribution, strawberries in CA maintained significantly lower respiration rates than those in the Reefer container (*p* < 0.05). The respiration rate of fruits is inhibited under conditions of low O_2_ and high CO_2_ concentrations compared with that in ambient air [35]. CO_2_ inhibits TCA cycle enzymes such as succinate dehydrogenase (SDH), leading to the accumulation of succinic acid, which delays respiration. High CO_2_ also inhibits pyruvate dehydrogenase (PDH), which catalyzes the conversion of pyruvate to acetyl-CoA, suppressing early respiration [35]. Additionally, CO_2_ further inhibits glycolytic enzymes, lowering ATP synthesis and, ultimately, respiration [36].

The CA treatment significantly reduced fruit decay at both maturity stages (Figure 1d). High CO_2_ concentrations (10–20%) can suppress *Botrytis cinerea*, the primary spoilage mold in strawberries [37]. CO_2_ lowers intracellular pH by dissolving into fruit tissues, inhibiting enzyme synthesis and activity, thereby reducing mold growth [33,34].

**Figure 2:**
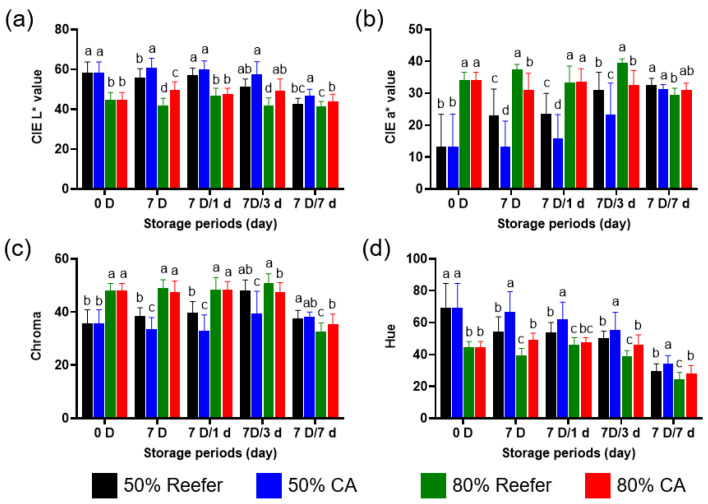
Comparison of the effect of controlled atmosphere (CA; 5% O_2_ + 12% CO_2_) and Reefer (air) container treatments on skin color index (CIE L* (**a**), CIE a* (**b**), hue angle (**c**), and chroma (**d**)) of strawberries. The strawberries were harvested (0 D) at different maturity stages (50 and 80%), containerized for 7 days (7 D) at 4 °C, and then subjected to cold distribution for 7 days (7 D/1 d, 7 D/3 d, and 7 D/7 d) at 10 °C. Data are presented as means ± standard deviations. Different letters indicate statistically significant differences.

The color attributes of Reefer- and CA-containerized strawberries are illustrated in Figure 2. The CIE L* value, which indicates brightness, initially showed a distinct difference between strawberries at 50% and 80% maturity (Figure 2a). However, after 3 d (7 D/3 d) of cold distribution, the CIE L* value for the 80%-maturity CA treatment group was not significantly different from that of the 50%-maturity Reefer and 50%-maturity CA treatment groups (*p* < 0.05). This trend continued through 7 days, while 80%-maturity fruit under the Reefer treatment group became slightly darker as the ripening progressed. The CIE a* value is used to predict the maturity level of strawberries. Initially, 50%- and 80%-maturity groups significantly differed in redness (*p* < 0.05); however, 50%-maturity strawberries turned redder over time (Figure 2b). By 7 days (7 D/7 d) of distribution, no difference was evident between the treatments. The CIE a* value notably increased in the 50%-maturity Reefer treatment group, whereas no such increase was observed in the 80%-maturity Reefer group. The CA treatment also delayed the ripening. Similar patterns were found in the hue angle (color tone) and chroma (saturation). A smaller hue angle indicates a redder color. During the storage, a significant difference was noted between the Reefer and CA treatments (*p* < 0.05) (Figure 2c). The hue angle decreased more rapidly under Reefer conditions, indicating faster reddening than under CA.

When comparing the different maturity stages, a significant difference was noted between the 50% and 80% maturity levels in the early stages (*p* < 0.05). However, after 7 d (7 D) in the CA container, the hue angle of the 50%-maturity Reefer-treated strawberries was not significantly different from that of the 80%-maturity CA-treated strawberries. This persisted throughout distribution, suggesting that the 50%-maturity fruit in Reefer can visually match the 80%-maturity fruit in the CA container. At 80% maturity, chroma values were similar between treatments early and up to the first day (7 D/1 d). However, at 50% maturity, we observed a significant difference between the Reefer and CA treatments (*p* < 0.05) (Figure 2d). After 3 d (7 D/3 d) of cold distribution, the differences between the container conditions exhibited a trend like that for the hue angle, suggesting that a slightly advanced harvest timing could improve flavor in less mature fruit without compromising its appearance during the Reefer export.

Korean strawberries exported to Southeast Asia, such as Vietnam and Hong Kong, are typically harvested before 50% maturity to allow ripening during transit. However, as non-climacteric fruits, strawberries fail to fully develop their unique flavor and aroma when ripened off the plant, which reduces consumer satisfaction and may negatively affect the brand reputation of Korean strawberries over the long term [9].

### 3.2. Multivariate Statistical Analysis of Metabolites According to the Maturity Stage and Container Environment

**Figure 3:**
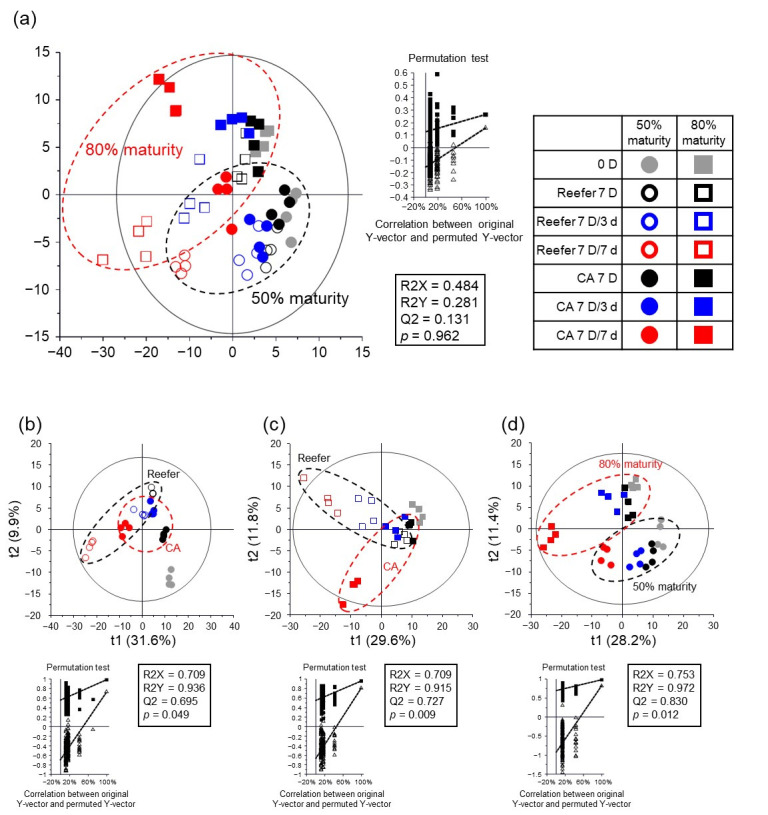
(**a**) PLS-DA score plot of strawberry metabolites at different maturity stages under the CA and Reefer during simulated export; (**b**) strawberries at 50% maturity; (**c**) strawberries at 80% maturity; (**d**) strawberries under the CA condition. Symbols indicate maturity stages (circle, 50% maturity; box, 80% maturity), export methods (open symbols, Reeder; filled symbols, CA), and export periods (gray, 0 D; black, 7 D; blue, 7 D/3 d; red, 7 D/7 d).

The metabolite profiles of strawberries were analyzed using GC-MS and UPLC-QTOF MS, and the statistical distinction between samples was visualized using PLS-DA score plots (Figure 3). Regarding the PLS-DA score plots for all sample groups, no significant correlation (*p* > 0.05) was observed in the permutation plot (Figure 3a). In contrast, the PLS-DA score plots for each group (50% maturity, 80% maturity, and CA) were statistically acceptable based on the goodness of fit (R^2^X = 0.709–0.753; R^2^Y = 0.915–0.972), predictability (Q^2^ = 0.695–0.830), *p*-values (<0.05), and cross-validation of the permutation test (*n* = 200).

In the score plots, strawberry samples were separated based on the Reefer and CA storage at 50% and 80% maturity (Figure 3b,c). In addition, the PLS-DA score plot for CA was also clearly separated according to maturity (Figure 3d). The variable importance in the projection (VIP) and *p*-values of the metabolites were analyzed in order to identify the metabolites that contributed to the separation between the sample groups in the PLS-DA score plots (Tables S1 and S2). A total of 115 metabolites, including primary (sugars, acidic compounds, and amino acids) and secondary metabolites (VOCs, terpenoids, and phenolic compounds), were identified based on the VIP (>0.7) and *p*-value (<0.05) (Tables S1 and S2). Similar metabolites were detected in a previous metabolomic study on strawberries [38].

Metabolomics is a powerful tool for postharvest quality assessment and understanding metabolic regulation in response to external stimuli [38,39]. In this study, PLS-DA score plots distinguished strawberry samples by maturity stage and storage condition (Figure 3). Although the overall model (Figure 3a) showed limited predictive value (Q^2^ = 0.131 and *p* = 0.962), individual models for 50% and 80% maturity (Figure 3b,c) demonstrated high reliability, especially in the 80% maturity group (R^2^Y = 0.915, Q^2^ = 0.727, and *p* = 0.009). The strongest separation was observed between maturity stages under CA conditions (Figure 3d; R^2^Y = 0.972, Q^2^ = 0.830, and *p* = 0.012), highlighting the maturity-dependent metabolic responses to storage environments.

### 3.3. Primary and Secondary Metabolites According to the Maturity Stage and Container Environment

**Figure 4:**
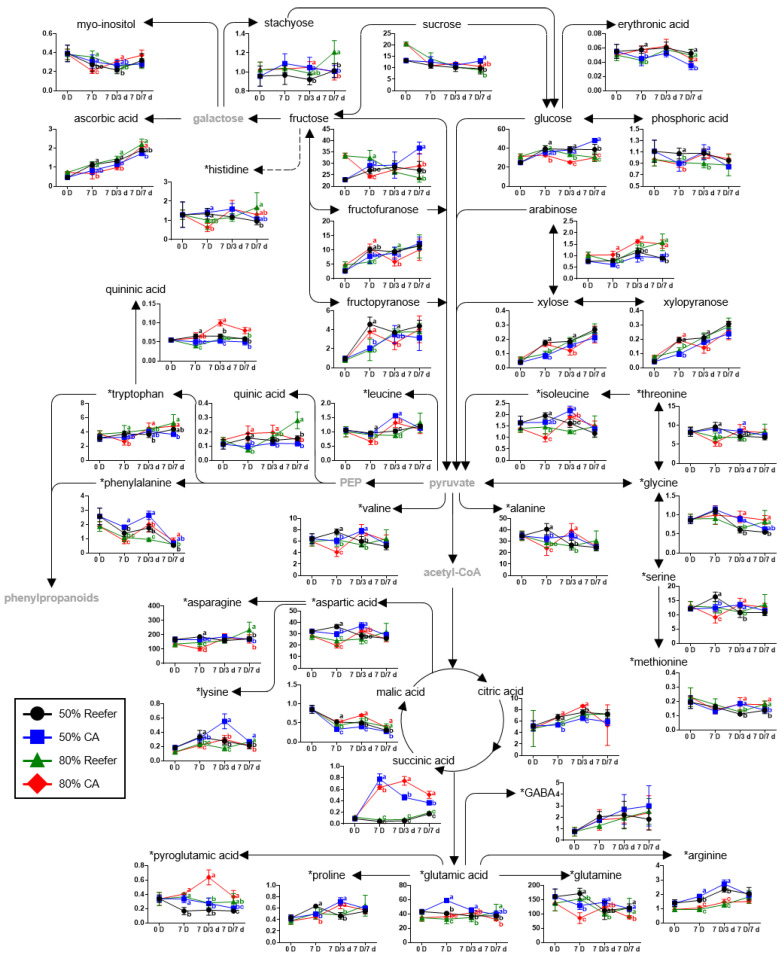
Metabolomic pathways of primary metabolites of strawberry according to the effect of controlled atmosphere (CA; 5% O_2_ + 12% CO_2_) and Reefer (air) container treatments. The strawberries were harvested (0 D) at different maturity stages (50 and 80%), containerized for 7 days (7 D) at 4 °C, and then subjected to cold distribution for 7 days (7 D/1 d, 7 D/3 d, and 7 D/7 d) at 10 °C. The *y*-axis represents relative abundance, calculated as normalized chromatogram intensity over internal standard intensity, while the *x*-axis indicates storage duration. * Amino acids were quantitatively analyzed with authentic standards. Data are expressed as means ± standard deviations. Different letters indicate statistically significant differences.

**Figure 5:**
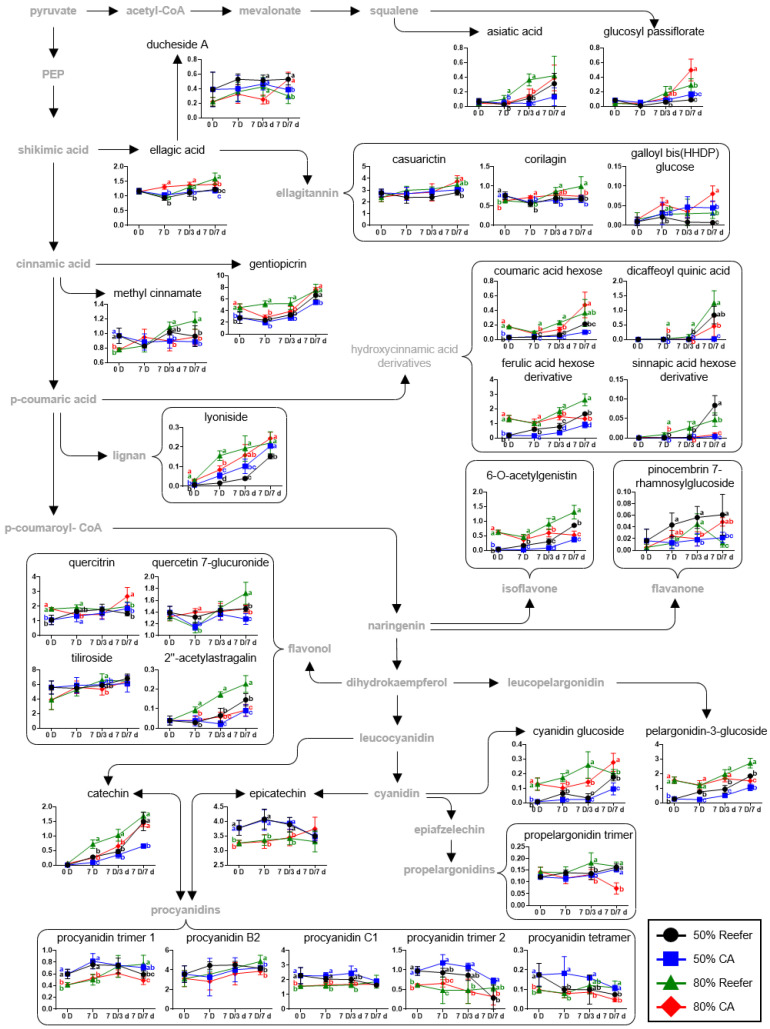
Metabolomic pathways of secondary metabolites of strawberry according to the effect of controlled atmosphere (CA; 5% O_2_ + 12% CO_2_) and Reefer (air) container treatments. The strawberries were harvested (0 D) at different maturity stages (50 and 80%), containerized for 7 days (7 D) at 4 °C, and then subjected to cold distribution for 7 days (7 D/1 d, 7 D/3 d, and 7 D/7 d) at 10 °C. The *y*-axis represents relative abundance, calculated as normalized chromatogram intensity over internal standard intensity, while the *x*-axis indicates storage duration. Data are expressed as means ± standard deviations. Different letters indicate statistically significant differences.

**Figure 6:**
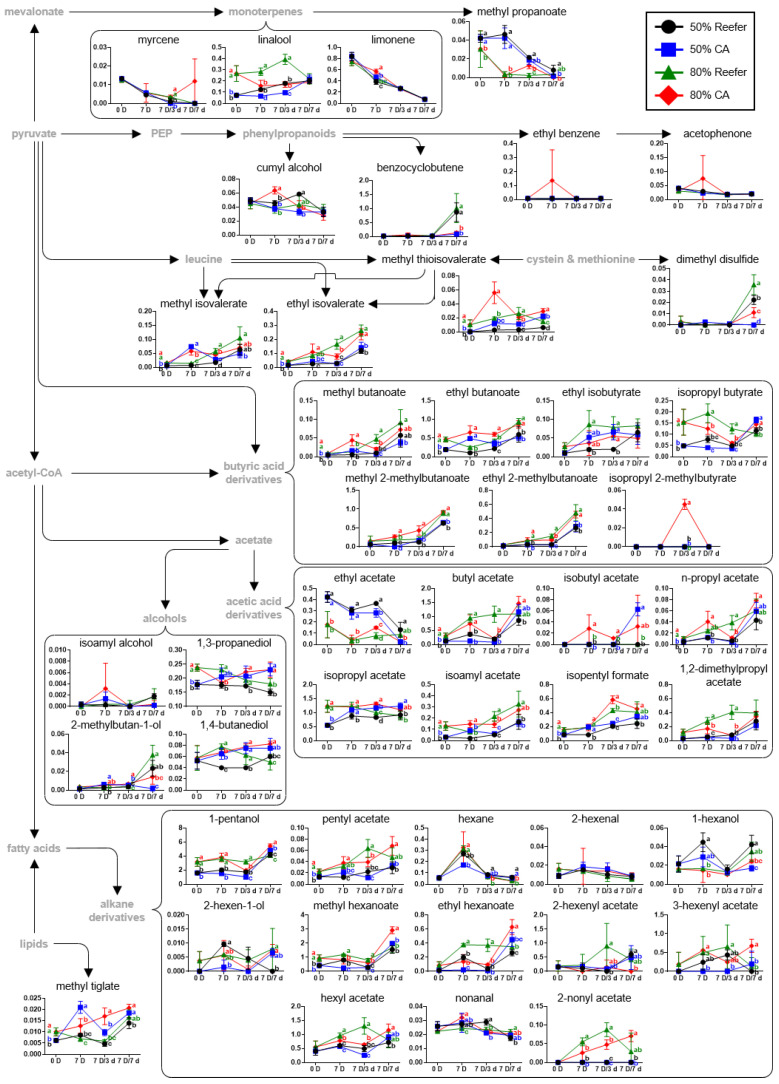
Metabolomic pathways of volatile organic compounds (VOCs) of strawberry according to the effect of controlled atmosphere (CA; 5% O_2_ + 12% CO_2_) and Reefer (air) container treatments. The strawberries were harvested (0 D) at different maturity stages (50 and 80%), containerized for 7 days (7 D) at 4 °C, and subjected to cold distribution for 7 days (7 D/1 d, 7 D/3 d, and 7 D/7 d) at 10 °C. The *y*-axis represents relative abundance, calculated as normalized chromatogram intensity over internal standard intensity, while the *x*-axis indicates storage duration. * Amino acids were quantitatively analyzed with authentic standards. Data are expressed as means ± standard deviations. Different letters indicate statistically significant differences.

Based on the identified metabolites (Tables S1 and S2), a strawberry metabolomic pathway was proposed according to the container conditions and distribution periods, and the relative abundances, calculated by dividing the chromatogram intensity by the IS intensity, of the identified metabolites were compared (Figure 4, Figure 5 and Figure 6).

The CA container treatment altered the primary and secondary metabolite levels in the 50%- and 80%-maturity strawberries compared to the treatment with Reefer containers.

Among the primary metabolites (Figure 4), the levels of major sugars, including sucrose, fructose, and glucose, at 0 d (0 D) were higher in strawberries at 80% than 50% maturity, whereas fructose and glucose levels increased during storage in strawberries at 50% maturity. In particular, the levels of fructose and glucose at 50% maturity in the CA container after 7 d (7 D/7 d) of cold distribution increased by 60% and 90%, respectively, compared with those at harvest. Additionally, xylose and arabinose were detected; however, the xylose content did not show a distinct trend according to the maturity or container type. In contrast, the arabinose content differed significantly depending on the maturity level. After 3 d (7 D/3 d) of cold distribution, strawberries at 50% maturity subjected to CA treatment had the lowest arabinose content, whereas those at 80% maturity subjected to CA treatment had the highest content (*p* < 0.05). After 7 d (7 D/7 d), no significant differences were evident between the treatments (*p* < 0.05). The levels of major acidic compounds, such as citric acid, malic acid, and ascorbic acid, showed fewer differences between the sample groups according to the maturity and container conditions. On the contrary, succinic acid levels showed less change in Reefer but a significant increase after 7 d (7 D) under CA conditions, being 19.4- and 9.7-times higher than those in Reefer at 50% and 80% maturities, respectively. The content of some amino acids, namely, phenylalanine, leucine, isoleucine, glycine, lysine, glutamic acid, proline, pyriglutamic acid, and proline, was higher in the CA treatment group after 7 d cold distribution (7 D/7 d).

The sensory quality of fruit is determined by a complex interplay of factors, such as the taste, aroma, color, and texture, with the content of primary metabolites, such as sugars and acids, as well as the sugar–acid ratio, significantly influencing consumer preferences [40]. During postharvest distribution, fruit respiration directly affects major metabolic pathways, such as sugar metabolism, starch metabolism, and the TCA cycle, leading to changes in the sugar, amino acid, and organic acid levels. Fruits use carbohydrates, organic acids, proteins, and fats as primary respiratory substrates during storage [15]. Low O_2_ conditions affect the enzyme activity and gene expression related to sugar metabolism in fruits. In apples, the reduction in sucrose content proceeds more slowly under CA storage than in air because of the low invertase and high sucrose synthase activity [41,42]. The expression of the sucrose synthase gene is highly induced in apples stored under low O_2_ conditions, which activates an alternative pathway using inorganic pyrophosphate to compensate for the ATP deficiency [16]. A 20 kPa CO_2_ treatment tended to maintain higher sucrose levels than the controls, suggesting that CO_2_ treatment can mitigate the loss of sweetness related to postharvest distribution [42].

The strawberry taste is primarily determined by the sucrose content and total volatile compounds, two factors that can be reduced by temperature changes, a water deficit, or changes in the CO_2_ concentration, negatively affecting the strawberry taste [9]. Sucrose has been proposed as the most critical metabolite contributing to the overall taste of strawberries because it directly determines the sweetness of strawberries and can enhance their aroma by interacting with volatile compounds [9].

In addition to the primary sugars, namely, sucrose, glucose, and fructose, fruits contain small amounts of other sugars and sugar alcohols, such as sorbitol, galactinol, raffinose, myo-inositol, and trehalose. Despite being present at low concentrations, these compounds are essential for mitigating the adverse effects of abiotic stress during postharvest storage [43]. After 3 d (7 D/3 d) of cold distribution following the container treatment, the myo-inositol content in the CA treatment group was significantly higher than that in the Reefer group, indicating the increased alleviation of abiotic stress, such as low O_2_ and high CO_2_ under CA conditions (*p* < 0.05).

Postharvest softening is a natural physiological process; excessive softening increases the susceptibility to damage and decay, compromising the fruit quality. Softening is mainly caused by cell wall degradation and can be monitored by measuring primary metabolites, such as monosaccharides, xylose, galactose, arabinose, and mannose [44].

Changes in the organic acid content during cold distribution are affected by various factors, such as the commodity type, storage temperature, and postharvest treatment before storage. For example, the content of malic and quinic acids decreases during cold storage, and these acids are eventually converted to secondary metabolites, such as anthocyanins, through subsequent processes [45]. In this study, malic acid declined during storage but remained higher under CA, particularly at 80% maturity (*p* < 0.05). Organic acids aid fruits in adapting to low O_2_ stress. The decrease in the organic acid concentration is an adaptation for reducing the nitrogen metabolism to produce the substrates necessary for glycolysis. SDH converts succinic acid into fumaric acid during the TCA cycle. The FADH2, thus generated, contributes to ATP synthesis via the electron transport chain [46,47]. Under conditions of O_2_ deficiency, the efficiency of the TCA cycle and electron transport chain decreases, leading to the accumulation of succinic acid.

Under low O_2_ conditions, fruits activate fermentation to sustain respiration, which significantly alters the nitrogen metabolism and promotes alanine accumulation [48,49]. In this study, alanine levels increased after 3 days of cold storage following CA treatment (Figure 4). Several amino acids—including glutamic acid, proline, isoleucine, and serine—also increased under CA conditions, consistent with previous findings under hypoxic or high CO_2_ environments [39,50]. Proline, in particular, showed a significant increase, suggesting a cell-protective response to stress. The observed increases in proline, glutamic acid, and methionine after CA storage highlight their potential roles in enhancing stress tolerance. These changes may reflect mitochondrial inhibition under low O_2_, which shifts the energy metabolism toward glycolysis and fermentation while conserving ATP [16,49].

Changes in the content of secondary metabolites, including terpenoids, phenolic acids, anthocyanins, flavonoids, and tannins, were also observed (Figure 5). The content of most secondary metabolites, except for ducheside A, epicatechin, and procyaninids, was increased during cold storage. Among them, the content of glucosyl passiflorate increased sharply by 12.9 times that at the harvest in samples with 80% maturity subjected to CA treatment and cold distribution. Similarly, the level of galloyl bis (HHDP) glucose, an ellagitannin, was the highest in 80%-maturity samples under CA treatment and cold distribution. The significant increase in the content of glucosyl passiflorate and galloyl bis(HHDP) glucose under CA storage suggests that the controlled atmosphere effectively increased the levels of these secondary metabolites. Previous proteomics studies have also reported that the expression of enzymes, such as leucoanthoxyanidin dioxygenase, caffeic acid 3-O-methyltransferase, and chalcone-flavone isomerase, which peaked under CA conditions (2% O_2_ and 12% CO_2_) during the storage of strawberries, increased the biosynthesis of phenylpropanoids [18].

In contrast, the lyoniside (lignin) levels showed different trends depending on the maturity and distribution conditions, being higher in strawberries at 50% maturity subjected to CA treatment and in those at 80% maturity subjected to Reefer treatment. The content of hydroxycinnamic acid derivatives and flavonoids was generally higher in the Reefer treatment group than in the CA group, regardless of maturity. Specifically, among the hydroxycinnamic acid derivatives, the content of dicaffeoyl quinic acid and cinnapic acid hexose derivative showed a sharp increase in the Reefer group after 7 d (7 D/7 d) of cold distribution. This suggests that non-CA conditions are more favorable for accumulating certain phenolic compounds, which are known for their antioxidant properties [20]. Similar results have been reported in a previous metabolomics study on strawberries, in which some hydroxycinnamic acid derivatives, including dicaffeoylquinic acid, sinapic acid hexose derivative, and ferulic acid hexose derivative, significantly accumulated during low-temperature storage [38]

Among the flavonoids, there was less accumulation of anthocyanins under CA conditions, and no difference in epicatechin content was noted between Reefer and CA treatments until 3 d (7 D/ 3d) of cold distribution. For condensed tannins, the trends varied depending on the tannin type. Procyanidin trimers and tetramers were higher in the Reefer group during storage, whereas pelargonidin trimers were higher in the Reefer group at 80% maturity after 3 d of cold distribution. Pelargonidin-3-glucoside was the major anthocyanin, whereas cyanidin-3-glucoside was present in smaller amounts. The CA environment delayed the increase in anthocyanin accumulation, particularly pelargonidin-3-glucoside, and this delay was consistent for the 50%- and 80%-maturity levels. For 80% maturity, the anthocyanin content at harvest remained constant during CA treatment and cold distribution, while, in Reefer samples, it increased continuously. Similar trends were observed at 50% maturity. Although cyanidin-glucoside also increased during cold distribution, its accumulation appeared to be less affected by CA treatment. Previous studies have shown that high CO_2_ concentrations delay anthocyanin accumulation by inhibiting chlorophyllase and downregulating the expression of FaChl b reductase, FaPAT, and FaRCCR, as well as PAL, C4H, and 4CL [19,51].

Polyphenols and terpenoid compounds, the major secondary metabolites in fruits, contribute to the color, sensory, nutritional properties, and stress responses. The most abundant anthocyanin in strawberries is pelargonidin-3-glucoside, while others, such as cyanidin-3-glucoside, pelargonidin-3-rutinoside, pelargonidin-3-arabinoside, and cyanidin-3-rutinoside, are present in smaller amounts [19]. Cold exposure significantly increases the anthocyanin accumulation in anthocyanin-rich fruits, such as oranges and some plum varieties [19,42,52,53,54]. In contrast, high CO_2_ concentrations induce phenolic compound precursors to form proanthocyanidins instead of anthocyanins. Proanthocyanidin B3 significantly increased under high CO_2_, while catechin and procyanidin levels decreased in fruits not exposed to CO_2_, suggesting a metabolic shift toward anthocyanin biosynthesis [19]. Flavan-3-ol monomers such as catechin and epicatechin function in plant defense against stress and protect them from pathogens [19,55]. In Geumsil strawberries, CA treatment increased epicatechin levels at 50% maturity and catechin at 80%, with differences diminishing during distribution. Previous studies have shown that 20% CO_2_ treatment increases the catechin content in strawberries by up to 1.54 times compared to that at the initial harvest after 3 d of exposure to high CO_2_ concentrations [19]. Furthermore, 20% CO_2_ treatment increased catechin and procyanidin levels without reducing anthocyanins, indicating no pathway competition. Such responses may contribute to fungal decay resistance [19].

### 3.4. Analysis of VOCs According to Maturity Stage and Container Environment

Strawberries contain a diverse array of VOCs, among which esters are the most abundant, followed by alcohols, aldehydes, and ketones [22]. The content of major esters such as methyl butanoate, ethyl butanoate, and ethyl 2-methylbutanoate was higher in strawberries at 80% maturity, while methyl hexanoate and ethyl hexanoate were initially low under CA conditions but were maintained at significantly higher levels after 7 days of cold distribution. CA storage also increased methyl tiglate, which contributes to freshness (*p* < 0.05). While VOC levels were initially lower in CA-treated fruit, their relative abundance increased over time compared to the Reefer group, suggesting that CA conditions are effective in preserving or enhancing aroma profiles during storage. Strawberry aroma results from a mixture of esters, alcohols, and alkanes, formed via metabolic changes during ripening. Lipids and amino acids serve as precursors, generating aldehydes, alcohols, and, finally, esters via alcohol acyltransferases [56,57,58]. Alanine, which increased during cold distribution under CA treatment (Figure 3), is involved in ethyl ester biosynthesis, contributing to the sweet aroma. Under prolonged high CO_2_ conditions, fermentation leads to the accumulation of ethanol and acetaldehyde [59]. However, alcohols such as 1-hexanol and 2-methylbutan-1-ol remained lower under CA, while linalool—a terpene with sweet floral notes—was better preserved during distribution [60]. Similarly, 1,3-propanediol and 1,4-butanediol levels remained stable or increased under CA, in contrast to the declining trend observed in Reefer.

CA treatment led to preserving consumer-preferred VOCs, such as ethyl hexanoate and hexyl acetate, particularly in strawberries harvested at 80% maturity [58,61]. In contrast, VOCs associated with off-flavors were less prominent. Since VOC loss during cold storage is often linked to chilling injury and impaired biosynthesis [62], CA conditions appear to mitigate this degradation. Notably, the accumulation of flavor-active VOCs is most pronounced after the red 1/2 to 3/4 maturity stages [63], whereas strawberries harvested at 50% maturity lack fully developed aroma components [9,60]. Additionally, although sensory evaluation was not conducted in this study, previous studies have reported a substantial correlation between volatile compounds, including ethyl butanoate, ethyl hexanoate, and methyl butanoate, and consumer perceptions regarding the sweetness and flavor of strawberries [9,61]. These compounds were better retained or recovered under CA conditions during simulated export, which suggests a potential contribution to maintaining the sensory quality of strawberries.

### 3.5. Pathway Analysis According to Maturity and Container Environment

A pathway analysis revealed that CA storage modulated several metabolic routes potentially linked to aroma formation, including phenylalanine metabolism, alanine, asparagine, and glutamate metabolism, as well as starch and sucrose metabolism (Figure S2). Although classical volatile biosynthesis pathways were not directly enriched, the observed changes suggest that CA conditions influence aroma development indirectly through the regulation of upstream precursor metabolism. In particular, phenylalanine metabolism and anthocyanin biosynthesis—both enriched under CA conditions—are involved in the production of secondary metabolites related to flavor and pigmentation. Similar metabolic trends have been reported in strawberries, where modified atmosphere storage altered carbon and nitrogen metabolism, thereby affecting the volatile composition and preserving sensory quality during postharvest storage [8,64].

### 3.6. Bioluminescence-Based Verification of Lipid Peroxidation and Correlation Between Secondary Metabolites and Biophoton Emission Values According to Maturity Stage and Container Environment

**Figure 7:**
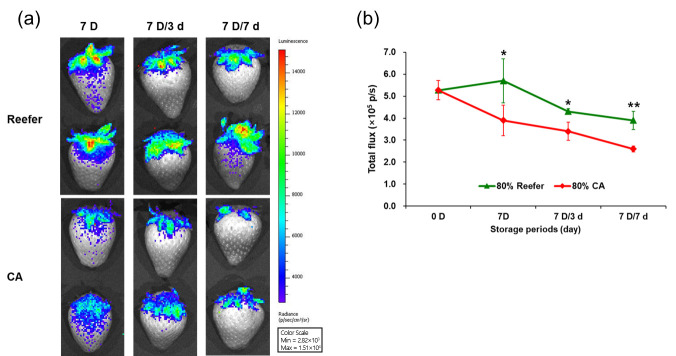
Comparison of the effect of controlled atmosphere (CA; 5% O_2_ + 12% CO_2_) and Reefer (air) container treatments on the intensity (**a**) and total flux (**b**) of bioluminescence emitted by strawberries. The strawberries were harvested (0 D) at different maturity stages (50 and 80%), containerized for 7 days (7 D) at 4 °C, and subjected to cold distribution for 7 days (7 D/1 d, 7 D/3 d, and 7 D/7 d) at 10 °C. Images were taken using a charge-coupled device camera-equipped In vivo Imaging System and analyzed using the Living image 4.7.2 software that associated a pseudo color scale to the emitted photon and assigned quantified (cps) values to the detected photon intensities shown on the right side of images. Data are expressed as means ± standard deviations. * *p* < 0.05, and ** *p* < 0.01 (*n* = 8).

The bioluminescence of strawberry samples at 80% maturity was measured nondestructively under the CA and Reefer container treatments (Figure 7). The highest biophoton emission values are shown in red, whereas the lowest values are shown in blue. Biophoton emission was higher in the Reefer group than in the CA group. When the total flux values for bioluminescence were measured to assess statistical significance, a significant difference was found between the CA and Reefer treatments immediately after the containers were opened (7 d) (*p* < 0.05), and the difference became more pronounced during the cold distribution (7 D/3–7 d) (*p* < 0.01). These results indicate that the Reefer container treatment resulted in a higher ROS production and increased lipid peroxidation of cell membranes compared with the CA treatment.

The Pearson correlation coefficients between VOCs, secondary metabolites, and IVIS bioluminescence were analyzed (Table S3). Among the VOCs, n-hexane, isopropyl butyrate, and limonene showed positive correlations with IVIS bioluminescence (*r* > 0.5), whereas methyl tiglate, methyl isovalerate, methyl 2-methylbutanoate, 2-methylbuthyl-d-3-acetate, isobutyl acetate, isoamyl acetate, and ethyl 2-methylbutanoate were negatively correlated (*r* < −0.47). The secondary metabolites ellagic acid, quercetin 7-glucuronide, glucosyl passiflorate, pinocembrin 7-rhamnosylglucoside, and coumaric acid hexose negatively correlated with IVIS bioluminescence (*r* < −0.48). These results suggest that antioxidant flavonoids act as resistance factors against cell membrane lipid peroxidation. A previous study on the IVIS of mangoes reported that flavonoid accumulation enhanced antioxidant activity and reduced ROS, improving the chilling tolerance [65].

CA treatment enhances cold tolerance by increasing the ratio of the fatty acid double bonds and sucrose content [66]. The double bond index (DBI), which reflects the ratio of unsaturated to saturated fatty acids, is a key indicator of membrane fluidity, helping protect cellular functions under cold conditions [66]. Sucrose, as both an energy source and cryoprotectant, stabilizes membranes by preventing intercellular freezing [66]. A high DBI value also supports the biosynthesis of volatile aroma products via the fatty acid LOX pathway, contributing to the flavor quality and consumer acceptability [66,67]. Additionally, CA conditions can influence mitochondrial metabolism and ROS generation by modulating the O_2_ availability [47]. Although SDH is a potential source of ROS in plant mitochondria, low O_2_ conditions under a CA environment reduce electron leakage and ROD production by inhibiting SDH activity, leading to succinic acid accumulation and enhanced cold stress [47]. The accumulation of ROS, particularly superoxide anions, causes lipid peroxidation and membrane damage [26]. High CO_2_ levels significantly lower ROS levels in strawberries [13]. Similar effects have been reported in litchi and persimmons, where CA or short-term high CO_2_ treatments enhanced antioxidant enzyme activity and maintained membrane permeability [68,69]. This study also demonstrates that CA conditions applied for strawberry export shipping are optimal for minimizing respiration while inhibiting lipid peroxidation, resulting in beneficial effects on flavor and quality retention.

This study focused on the ‘Geumsil’ cultivar, a major strawberry variety exported from Korea, with the aim of addressing the quality deterioration issues encountered during actual export logistics. While this cultivar selection was intentional to reflect realistic export conditions, it also represents a limitation of the study. The use of fruit harvested from a single cultivar and a single production region may restrict the broader applicability of the findings to other cultivars or growing regions. Therefore, future studies involving multiple strawberry cultivars from diverse geographic origins are warranted in order to validate and extend the generalizability of these results.

## 4. Conclusions

This study demonstrates that controlled atmosphere (CA) storage at 5% O_2_ and 12% CO_2_ during maritime transport, followed by cold chain distribution at 10 °C, is an effective strategy for maintaining the postharvest quality of strawberries, particularly those harvested at higher maturity stages. Compared with conventional reefer storage, CA treatment significantly reduced weight loss and decay, maintained firmness, and lowered respiration rates. Furthermore, it favorably modulated key metabolites—such as sucrose, succinic acid, and proline—as well as the VOCs responsible for the desirable aroma, thereby enhancing both the sensory and nutritional quality.

Pathway and imaging analyses supported the physiological benefits of CA conditions, including reduced oxidative stress and delayed anthocyanin accumulation, which contributed to an improved shelf life and visual quality. Based on these findings, we recommend the adoption of CA storage systems by strawberry exporters aiming to minimize quality deterioration during long-distance maritime transport. This approach offers a practical and scientifically validated solution for maintaining product value and competitiveness in global markets.

## Data Availability

The original contributions presented in this study are included in the Supplementary Materials. Further inquiries can be directed to the corresponding authors.

## Supplementary Materials

The following supporting information can be downloaded at: https://www.mdpi.com/article/10.3390/foods14172959/s1, Figure S1: Classification of strawberries based on skin coloration. Left: fruit harvested at approximately 50% skin coloration (early maturity); Right: fruit harvested at approximately 80% skin coloration (advanced maturity); Figure S2: Pathway analysis of strawberry metabolites according to the effect of controlled atmosphere (CA; 5% O_2_ + 12% CO_2_) and Reefer (air) container treatment. Pathway analysis was conducted using MetaboAnalyst 5.0 (https://www.metaboanalyst.ca), based on normalized intensities of identified metabolites and the KEGG database. Larger and darker bubbles indicate greater pathway relevance; Table S1: Identification of major metabolites contributing to difference between sample groups by GC-MS; Table S2: Identification of major metabolites contributing to difference between sample groups by HPLC and UPLC-Q-TOF MS; Table S3: Pearson’s correlation coefficient between strawberry secondary metabolites and IVIS.

## Abbreviations

**CA**	controlled atmosphere
**VOCs**	volatile organic compounds
**MAP**	modified atmosphere packaging
**IVIS**	In Vivo Imaging System
**CCD**	charge-coupled device
**GC-MS**	gas chromatography mass spectrometry
**UPLC**	ultra-performance liquid chromatography
**QTOF**	quadrupole time-of-flight
**PG**	polygalacturonase
**PME**	pectin methylesterase
**PL**	pectate lyase
**PDH**	pyruvate dehydrogenase
**SDH**	succinate dehydrogenase
