# Supplementary material for: Impact of Harvest Maturity and Controlled Atmosphere on Strawberry Quality Under Simulated Export Conditions
Source: Foods. 2025 Aug 25;14(17):2959. doi: 10.3390/foods14172959 (PMC12428743; doi:10.3390/foods14172959)
Supplement: Supplementary file 1 [file foods-14-02959-s001.zip › foods-3759134-supplementary.pdf]

Supplementary Information

The Impact of Maturity and Biochemical Responses in a CA Environment for Optimizing Strawberry Quality during Maritime Export

Hyang Lan Eum[1†*], Ji-Hyun Lee[2†], Jeong Gu Lee[2], Min-Sun Chang[2], Kyung-Ran Do[2], Haejo Yang[2], Kang-Mo Ku[3], Dong-Shin Kim[1*]

[1]Hiland Agriculture Research Institute, National Institute of Crop and Food Science, Wanju 55365, Republic of Korea

[2]Postharvest Research Division, National Institute of Horticultural and Herbal Science, Wanju 55365, Republic of Korea

[3]Department of Plant Biotechnology, College of Life Sciences and Biotechnology, Korea University, Seoul, Republic of Korea

*Correspondence: Hyang Lan Eum, eumhl76@korea.kr; Dong-Shin Kim, dskim3309@korea.kr

†These authors have contributed equally to this work and share first authorship

E-mail address of authors:

Ji-Hyun Lee, leejh80@korea.kr; Jeong Gu Lee, ljg89@korea.kr; Min-Sun Chang, aeru@korea.kr; Kyung-Ran Do, microdo@korea.kr; Haejo Yang, gowh1231@korea.kr; Kang-Mo Ku, ku_km@korea.ac.kr

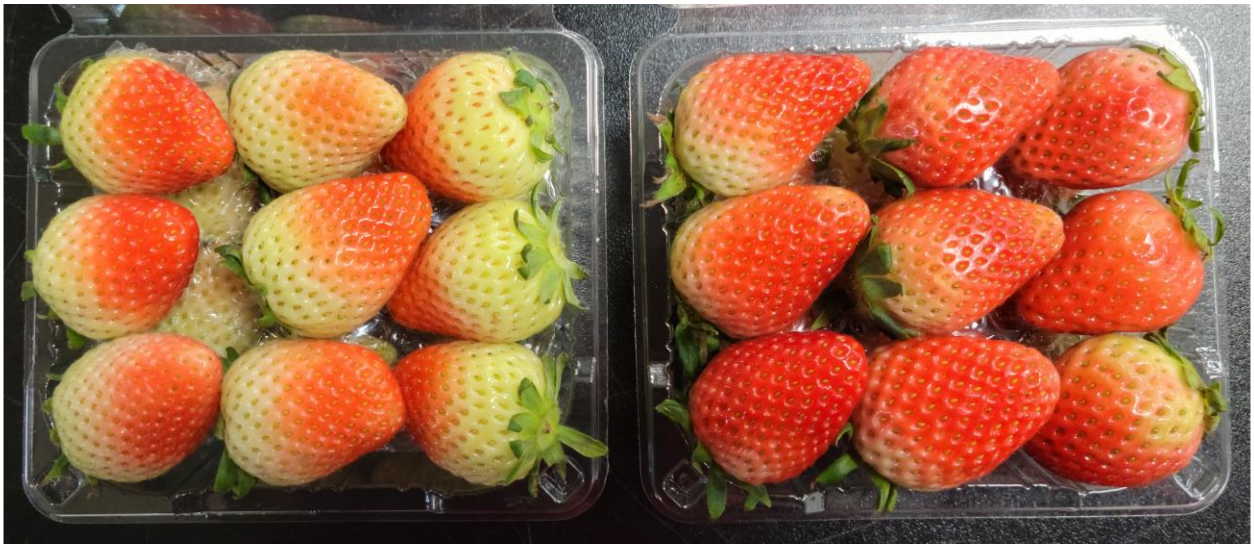

Figure S1. Classification of strawberries based on skin coloration. Left: fruit harvested at approximately 50% skin coloration (early maturity); Right: fruit harvested at approximately 80% skin coloration (advanced maturity).

Table S1. Identification of major metabolites contributing to the difference between sample groups using GC-MS.

	RT	Compounds	RI	50% maturity		80% maturity		50% CA *vs*. 80% CA	
				VIP	*p*-value	VIP	*p*-value	VIP	*p*-value
Volatile organic compounds	1.61	n-hexane	663	1.51	9.66×10^{-13}	1.48	7.94×10^{-6}	1.34	3.08×10^{-5}
	1.70	ethyl acetate	667	1.09	1.66×10^{-7}	1.29	2.29×10^{-2}	1.02	3.85×10^{-7}
	1.81	methyl propanoate	671	1.11	1.35×10^{-6}	1.32	3.71×10^{-3}	1.12	1.50×10^{-5}
	2.08	isopropyl acetate	682	1.31	2.41×10^{-6}	0.94	1.43×10^{-2}	1.41	3.33×10^{-6}
	2.70	ethyl propanoate	707	1.04	8.58×10^{-6}	1.07	9.18×10^{-5}	0.95	2.64×10^{-7}
	2.75	n-propyl acetate	710	0.77	7.67×10^{-4}	0.96	1.28×10^{-4}	0.93	2.20×10^{-7}
	2.89	methyl butanoate	717	1.09	5.50×10^{-11}	1.34	3.14×10^{-6}	1.05	2.19×10^{-10}
	3.16	1-pentanol	729	1.25	6.41×10^{-3}	1.05	3.64×10^{-1}	0.97	3.15×10^{-1}
	3.16	isoamyl alcohol	729	1.08	3.90×10^{-10}	1.06	4.16×10^{-7}	1.12	6.33×10^{-4}
	3.26	dimethyl disulfide	734	1.20	2.10×10^{-3}	1.11	7.24×10^{-2}	1.01	5.67×10^{-2}
	3.63	ethyl isobutyrate	751	0.89	1.16×10^{-7}	0.91	1.33×10^{-8}	0.89	3.00×10^{-10}
	3.63	methyl isobutyrate	751	1.46	3.45×10^{-10}	0.88	3.82×10^{-1}	1.08	2.48×10^{-2}
	3.98	isobutyl acetate	768	1.42	5.91×10^{-7}	0.98	1.60×10^{-4}	1.39	1.51×10^{-7}
	4.04	methyl isovalerate	771	1.11	5.41×10^{-5}	1.21	2.63×10^{-6}	0.95	5.26×10^{-6}
	4.66	ethyl butanoate	800	1.13	1.06×10^{-7}	1.16	6.99×10^{-5}	0.96	7.95×10^{-8}
	5.01	butyl acetate	813	0.76	7.43×10^{-5}	1.04	2.21×10^{-3}	0.86	7.72×10^{-7}
	5.41	1,2-dimethylpropyl acetate	827	1.50	3.27×10^{-13}	1.52	1.10×10^{-8}	1.49	4.50×10^{-11}
	5.78	isopropyl butyrate	840	1.28	6.83×10^{-10}	1.24	4.51×10^{-3}	1.16	3.69×10^{-6}
	5.97	ethyl 2-methylbutanoate	847	0.95	6.60×10^{-8}	0.89	4.12×10^{-8}	0.82	1.96×10^{-10}
	6.06	2-hexenal	850	1.08	1.61×10^{-2}	0.39	8.29×10^{-1}	0.80	8.23×10^{-1}
	6.11	methyl 2-methylbutanoate	852	1.00	4.64×10^{-12}	1.01	8.52×10^{-9}	0.82	5.13×10^{-10}
	6.11	ethyl isovalerate	852	0.96	1.09×10^{-6}	0.86	6.39×10^{-6}	0.81	1.28×10^{-6}
	6.17	ethylbenzene	854	1.05	7.44×10^{-3}	0.94	4.48×10^{-1}	0.93	4.50×10^{-11}
	6.37	methyl tiglate	861	1.39	1.87×10^{-8}	1.26	2.20×10^{-5}	1.52	1.54×10^{-6}
	6.54	1-hexanol	867	1.42	6.18×10^{-4}	1.08	5.77×10^{-3}	1.21	6.05×10^{-2}
	6.59	2-methylbutan-1-ol	869	1.09	3.04×10^{-5}	1.04	5.49×10^{-6}	1.05	2.98×10^{-3}
	6.74	isoamyl acetate	874	0.90	5.14×10^{-5}	0.87	6.48×10^{-4}	0.81	3.64×10^{-7}
	6.80	2-methylbutyl-d-3 acetate	876	1.21	8.55×10^{-7}	1.38	9.27×10^{-6}	1.22	7.62×10^{-10}
	7.01	isopropyl 2-methylbutyrate	884	NC	NC	1.70	7.22×10^{-13}	1.59	1.67×10^{-14}
	7.03	benzocyclobutene	884	0.93	6.12×10^{-6}	1.05	1.90×10^{-4}	0.83	4.19×10^{-3}
	7.79	pentyl acetate	912	1.09	1.14×10^{-3}	1.07	3.63×10^{-3}	0.82	6.56×10^{-6}
	8.09	methyl hexanoate	922	1.24	1.66×10^{-10}	1.14	7.57×10^{-8}	0.87	1.15×10^{-11}
	8.51	methyl thioisovalerate	936	1.32	3.95×10^{-8}	1.51	1.13×10^{-4}	1.34	1.11×10^{-6}
	9.94	myrcene	984	1.20	2.88×10^{-11}	1.12	6.26×10^{-2}	1.19	8.74×10^{-3}
	10.25	ethyl hexanoate	994	1.32	3.85×10^{-8}	1.15	1.87×10^{-6}	0.86	9.87×10^{-9}
	10.42	3-hexenyl acetate	1000	1.41	2.45×10^{-2}	0.93	2.64×10^{-1}	0.95	2.40×10^{-3}
	10.64	hexyl acetate	1008	1.30	7.31×10^{-5}	1.31	4.64×10^{-3}	1.03	1.53×10^{-2}
	10.72	2-hexenyl acetate	1010	0.89	8.71×10^{-3}	1.01	1.34×10^{-1}	1.23	1.22×10^{-1}
	11.04	limonene	1022	1.02	5.56×10^{-12}	1.06	3.28×10^{-12}	1.05	3.51×10^{-13}
	11.08	2-hexen-1-ol	1023	1.50	7.20×10^{-5}	1.01	3.56×10^{-1}	0.98	6.47×10^{-3}
	11.82	2-nonyl acetate	1049	NC	NC	1.39	9.98×10^{-3}	0.97	4.43×10^{-6}
	12.05	acetophenone	1057	1.36	1.55×10^{-5}	1.01	3.12×10^{-1}	0.99	2.97×10^{-1}
	12.64	cumyl alcohol	1078	1.55	4.92×10^{-5}	1.20	3.53×10^{-3}	1.21	6.73×10^{-5}
	13.06	linalool	1093	1.07	5.74×10^{-8}	1.50	1.64×10^{-4}	1.16	1.71×10^{-5}
	13.20	nonanal	1098	1.28	2.33×10^{-3}	1.49	1.77×10^{-5}	1.21	1.06×10^{-3}
Non-volatile compounds	15.86	1,3-propanediol	814	1.11	6.02×10^{-4}	1.20	1.64×10^{-4}	1.06	7.21×10^{-3}
	19.09	succinic acid	922	1.30	9.91×10^{-5}	1.03	1.17×10^{-2}	0.73	3.51×10^{-2}
	22.22	phosphoric acid	930	1.03	1.25×10^{-2}	0.96	1.29×10^{-1}	1.11	1.53×10^{-2}
	23.48	butanedioic acid	1315	1.49	5.72×10^{-17}	1.47	2.34×10^{-17}	1.54	3.14×10^{-16}
	27.90	malic acid	1475	1.24	4.56×10^{-13}	1.28	8.69×10^{-9}	1.20	4.16×10^{-13}
	28.73	pyroglutamic acid	1523	0.98	1.14×10^{-4}	1.36	9.14×10^{-5}	1.34	7.74×10^{-7}
	29.64	erythronic acid	1569	1.12	1.60×10^{-3}	1.18	4.68×10^{-3}	1.10	1.36×10^{-3}
	32.32	arabinose	1720	1.27	7.95×10^{-4}	1.12	5.69×10^{-6}	1.01	6.19×10^{-9}
	35.02	xylose	1728	0.87	1.71×10^{-12}	0.92	1.06×10^{-6}	1.00	2.13×10^{-9}
	37.38	xylopyranose	1734	0.86	5.98×10^{-13}	0.92	1.28×10^{-6}	1.02	1.49×10^{-9}
	39.50	fructofuranose	1775	1.02	2.44×10^{-8}	0.97	6.04×10^{-3}	1.13	1.41×10^{-6}

	39.71	fructopyranose	1786	1.03	6.15×10^{-6}	1.08	1.02×10^{-4}	1.10	2.59×10^{-5}
	40.14	citric acid	1798	1.13	2.53×10^{-5}	1.00	8.31×10^{-2}	0.97	8.28×10^{-2}
	41.59	quininic acid	1839	1.07	7.16×10^{-3}	1.45	7.30×10^{-9}	1.25	3.27×10^{-8}
	42.37	fructose	1875	1.13	3.41×10^{-4}	1.15	1.09×10^{-4}	1.32	3.45×10^{-5}
	43.35	glucose	1926	1.15	3.62×10^{-7}	1.29	8.14×10^{-5}	1.35	1.33×10^{-11}
	51.19	myo-inositol	2095	1.06	2.96×10^{-2}	1.25	4.63×10^{-4}	1.18	6.07×10^{-4}
	63.12	sucrose	2705	0.93	3.34×10^{-2}	1.24	9.41×10^{-9}	1.36	1.40×10^{-8}

RT, retention time; RI, retention indices; VIP, variable importance in the protection; CA, controlled atmosphere storage

Table S2. Identification of major metabolites contributing to the difference between sample groups using HPLC and UPLC-Q-TOF MS

	RT	Compounds	Adduct	Exact mass (m/z)	mass fragment	50% maturity		80% maturity		50% CA *vs.* 80% CA	
						VIP	p-value	VIP	p-value	VIP	p-value
HPLC	2.22	aspartic acid				1.54	1.14×10^{-6}	1.16	8.15×10^{-3}	1.29	3.61×10^{-7}
	3.88	glutamic acid				1.32	4.50×10^{-8}	0.91	1.23×10^{-1}	1.33	1.40×10^{-8}
	7.62	asparagine				1.29	8.38×10^{-2}	0.94	3.02×10^{-4}	1.20	8.73×10^{-5}
	8.21	serine				1.49	1.09×10^{-5}	1.11	5.02×10^{-2}	1.22	3.22×10^{-3}
	9.21	glutamine				1.40	1.66×10^{-3}	1.24	2.65×10^{-3}	1.01	5.70×10^{-5}
	9.85	histidine				0.71	1.40×10^{-1}	0.98	4.07×10^{-2}	1.10	1.86×10^{-2}
	10.27	glycine				1.20	1.47×10^{-10}	1.01	1.93×10^{-1}	1.29	1.68×10^{-3}
	10.49	threonine				1.05	1.38×10^{-3}	1.15	1.09×10^{-2}	1.35	7.65×10^{-4}
	11.29	arginine				1.46	4.28×10^{-6}	0.87	6.14×10^{-5}	1.31	4.09×10^{-11}
	12.38	alanine				1.28	6.29×10^{-6}	1.21	6.19×10^{-3}	1.16	6.25×10^{-5}
	12.92	GABA				0.84	1.14×10^{-1}	0.71	1.25×10^{-1}	0.80	3.60×10^{-2}
	16.82	valine				1.35	1.35×10^{-5}	1.33	6.31×10^{-3}	1.30	3.44×10^{-5}
	17.09	methionine				1.66	4.93×10^{-4}	1.08	2.86×10^{-2}	1.12	1.28×10^{-2}
	18.26	tryptophan				1.02	2.93×10^{-4}	0.95	1.29×10^{-3}	1.08	3.11×10^{-6}
	18.83	phenylalanine				1.14	8.57×10^{-10}	1.25	2.60×10^{-6}	1.21	7.44×10^{-9}
	19.14	isoleucine				1.21	1.51×10^{-6}	1.29	1.21×10^{-2}	1.30	6.27×10^{-6}
	20.04	leucine				1.83	6.71×10^{-12}	1.23	3.21×10^{-4}	1.48	5.86×10^{-9}
	20.71	lysine				1.42	1.16×10^{-6}	1.59	2.02×10^{-5}	1.41	7.16×10^{-10}
	25.18	proline				1.68	3.21×10^{-7}	1.23	1.95×10^{-3}	1.30	2.79×10^{-8}
UPLC-QTOF MS	0.73	quinic acid	M-H	191.0535	127	0.92	1.10×10^{-2}	1.11	1.79×10^{-5}	1.03	8.90×10^{-4}
	0.75	lupeose	M-H	683.2269	89, 191, 281, 323	0.88	1.78×10^{-1}	1.07	1.13×10^{-2}	0.80	5.40×10^{-1}
	0.81	ascorbic acid	M-H	175.022	87	0.84	8.53×10^{-13}	0.81	2.18×10^{-10}	0.84	2.77×10^{-12}
	2.55	procyanidin trimer 1	M-H	865.2021	287, 575	1.08	1.00×10^{-2}	0.96	9.84×10^{-4}	1.17	2.70×10^{-7}
	2.63	methyl cinnamate	M-H	368.0966	161, 131	1.10	1.04×10^{-1}	0.86	1.46×10^{-4}	0.96	2.36×10^{-1}
	2.78	catechin	M-H	289.0906	123, 137, 149	0.76	5.07×10^{-12}	0.84	1.39×10^{-11}	0.77	2.07×10^{-13}
	2.89	cyanidin glucoside	M-H	447.0918	285	0.85	1.35×10^{-8}	1.05	2.56×10^{-4}	0.80	5.52×10^{-10}
	2.97	procyanidin B2	M-H	577.1353	108, 125, 287, 289	0.73	4.92×10^{-1}	0.68	1.24×10^{-2}	0.66	5.92×10^{-1}
	2.97	corilagin	M-H	633.0739	275, 300, 481	1.04	3.67×10^{-2}	0.82	6.67×10^{-4}	1.34	2.76×10^{-3}
	3.00	procyanidin C1	M-H	865.2023	108, 125, 287, 289	0.77	2.94×10^{-2}	0.48	5.33×10^{-1}	0.91	5.88×10^{-4}
	3.01	ferulic acid hexose derivative	M-H	449.1075	431, 287, 269	0.77	8.06×10^{-13}	0.92	3.53×10^{-7}	0.98	1.52×10^{-10}
	3.01	pelargonidin-3-glucoside	M-H	431.0967	271	0.77	1.93×10^{-12}	0.93	1.07×10^{-6}	0.99	7.29×10^{-11}
	3.06	epicatechin	M-H	289.0697	123, 137, 149, 165	0.86	1.26×10^{-2}	0.61	2.23×10^{-1}	0.91	2.54×10^{-4}
	3.11	coumaric acid hexose	M-H	325.0907	145	0.80	3.04×10^{-15}	0.95	7.15×10^{-5}	0.88	7.97×10^{-9}
	3.13	procyanidin tetramer	M-H	1153.268	576, 707, 289	0.74	2.54×10^{-2}	1.01	1.15×10^{-2}	0.79	2.07×10^{-4}
	3.19	propelargonidin trimer	M-H	849.2074	561	0.78	1.30×10^{-3}	1.11	2.83×10^{-4}	1.23	7.12×10^{-5}
	3.21	procyanidin trimer 2	M-H	865.2019	243, 287, 559, 575	0.78	9.98×10^{-6}	0.75	5.12×10^{-1}	0.88	6.71×10^{-7}
	3.23	6-O-acetylgenistin	M-H	473.1078	268, 269	0.78	2.73×10^{-15}	0.95	2.56×10^{-7}	1.03	4.26×10^{-10}
	3.29	casuarictin	M-H	935.0842	125, 169, 275, 391	0.99	1.51×10^{-1}	0.68	1.53×10^{-2}	0.65	2.23×10^{-2}
	3.36	galloyl bis(HHDP) glucose	M-H	935.582	467, 431, 269	1.10	1.59×10^{-2}	1.01	4.00×10^{-3}	0.88	1.16×10^{-3}
	3.38	quercetin 7-glucuronide	M-H	447.0556	151, 299, 301	1.12	1.29×10^{-3}	0.96	2.95×10^{-5}	1.28	2.62×10^{-3}
	3.53	ellagic acid	M-H	300.9968		1.05	1.32×10^{-6}	1.04	2.46×10^{-6}	1.05	1.68×10^{-5}
	3.60	quercitrin	M-H	477.0664		0.90	4.13×10^{-2}	1.06	1.58×10^{-4}	0.86	1.11×10^{-4}
	3.76	ducheside A	M-H	447.0917	299	0.64	4.63×10^{-1}	0.98	8.48×10^{-3}	0.87	6.22×10^{-2}

3.88	2''-acetylastragalin	M-H	489.1031	227, 284	0.79	1.07×10^{-5}	0.92	1.16×10^{-9}	1.01	1.33×10^{-3}
3.91	gentiopicrin	M-H	355.1015	125, 147	0.84	3.57×10^{-10}	0.95	4.43×10^{-8}	0.84	3.60×10^{-11}
4.10	lyoniside	M-H	551.2341	165, 209	1.12	1.83×10^{-13}	0.97	3.82×10^{-6}	0.91	1.85×10^{-11}
4.28	tiliroside	M-H	593.1304		0.62	2.88×10^{-1}	0.86	4.06×10^{-3}	0.87	3.11×10^{-2}
4.74	glucosyl passiflorate	M-H	695.4028	487, 679	1.32	1.32×10^{-7}	0.99	7.64×10^{-8}	0.84	4.17×10^{-10}
5.02	sinnapic acid hexose derivative	M-H	385.1487	556	0.98	8.94×10^{-11}	0.96	2.72×10^{-6}	0.78	4.78×10^{-5}
5.61	dicaffeoyl quinic acid	M-H	561.2912	515, 191, 161	0.99	2.32×10^{-9}	0.98	1.04×10^{-7}	0.87	1.66×10^{-4}
6.05	asiatic acid	M+COO⁻	487.3419	425, 443, 469	0.74	5.07×10^{-4}	0.74	4.08×10^{-4}	0.76	7.06×10^{-5}
7.53	pinocembrin 7-rhamnosylglucoside	M-H	563.3806	255, 443	0.99	5.78×10^{-3}	1.29	1.97×10^{-5}	0.82	1.24×10^{-3}

RT, retention time; VIP, variable importance in the protection; CA, controlled atmosphere storage

Table S3. Pearson's correlation coefficient between strawberry secondary metabolites and IVIS.

Volatile organic compounds	IVIS	*p*-value	Phenolics and terpenoids	IVIS	*p*-value
n-hexane	0.63	**	propelargonidin trimer	0.25	ns
isopropyl butyrate	0.61	**	procyanidin C1	0.21	ns
limonene	0.50	*	procyanidin tetramer	0.12	ns
linalool	0.40	ns	2''-acetylastragalin	0.07	ns
dimethyl disulfide	0.38	ns	sinnapic acid hexose derivative	0.05	ns
2-hexenal	0.32	ns	lupeose	-0.04	ns
nonanal	0.27	ns	procyanidin trimer	-0.05	ns
acetophenone	0.21	ns	6-O-acetylgenistin	-0.12	ns
1-pentanol	0.20	ns	procyanidin C1	-0.13	ns
ethylbenzene	0.18	ns	procyanidin B2	-0.14	ns
isopropyl acetate	0.13	ns	dicaffeoyl quinic acid	-0.18	ns
3-hexenyl acetate	0.12	ns	epicatechin	-0.20	ns
cumyl alcohol	0.12	ns	ferulic acid hexose derivative	-0.21	ns
butyl acetate	0.09	ns	gentiopicrin	-0.22	ns
2-hyxenyl acetate	0.09	ns	pelargonidin-3-glucoside	-0.23	ns
hexyl acetate	0.04	ns	catechin	-0.24	ns
ethyl butanoate	-0.04	ns	methyl cinnamate	-0.24	ns
benzocyclobutene	-0.08	ns	ducheside A	-0.24	ns
ethyl hexanoate	-0.11	ns	asiatic acid	-0.27	ns
methyl thioisovalerate	-0.11	ns	ascorbic acid	-0.28	ns
2-nonyl acetate	-0.11	ns	quercitrin	-0.29	ns
2-hexen-1-ol	-0.13	ns	casuarictin	-0.29	ns
methyl propanoate	-0.15	ns	lyoniside	-0.30	ns
methyl butanoate	-0.20	ns	cyanidin glucoside	-0.33	ns
myrcene	-0.21	ns	corilagin	-0.37	ns
1-hexanol	-0.23	ns	quinic acid	-0.39	ns
isoamyl alcohol	-0.24	ns	tiliroside	-0.41	ns
ethyl acetate	-0.25	ns	galloyl bis(HHDP) glucose	-0.41	ns
isopropyl 2-methylbutyrate	-0.26	ns	coumaric acid hexose	-0.48	*
2-methylbutan-1-ol	-0.29	ns	pinocembrin 7-rhamnosylglucoside	-0.49	*
methyl isobutyrate	-0.30	ns	glucosyl passiflorate	-0.55	*
pentyl acetate	-0.38	ns	quercetin 7-glucuronide	-0.57	**
1,2-dimethylpropyl acetate	-0.38	ns	ellagic acid	-0.61	**
ethyl isovalerate	-0.40	ns			
methyl hexanoate	-0.41	ns			
ethyl propanoate	-0.41	ns			
n-propyl acetate	-0.44	ns			
ethyl isobutyrate	-0.45	ns			
ethyl 2-methylbutanoate	-0.47	*			
isoamyl acetate	-0.50	*			
isobutyl acetate	-0.50	*			
2-methylbutyl-d-3 acetate	-0.58	*			
methyl 2-methylbutanoate	-0.62	**			
methyl isovalerate	-0.66	**			
methyl tiglate	-0.73	***			

* $p < 0.05$, ** $p < 0.01$, *** $p < 0.001$

The colors of the correlation heat map represent the correlation coefficients; blue and red on a red–blue color scale indicate positive and negative correlations, respectively.

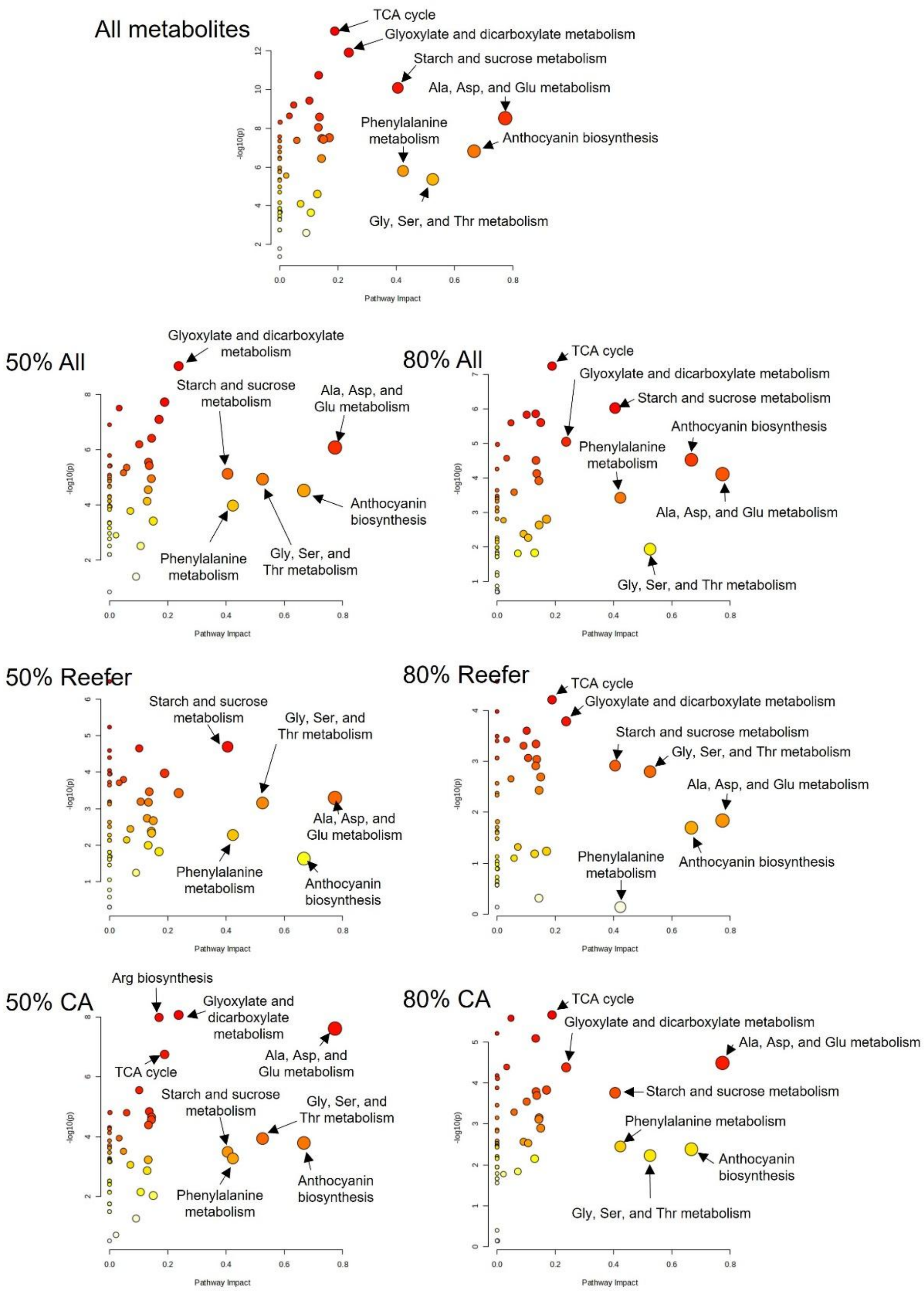

Figure S2. Pathway analysis of strawberry metabolites according to the effect of controlled atmosphere (CA; 5% O_2 + 12% CO_2) and Reeder (air) container treatment. Pathway analysis was conducted using MetaboAnalyst 5.0 (https://www.metaboanalyst.ca), based on normalized intensities of identified metabolites and the KEGG database. Larger and Darker bubbles indicate greater pathway relevance.
